# Editorial: Cell-Based Approaches for Modulating Cartilage and Bone Phenotype

**DOI:** 10.3389/fbioe.2021.716323

**Published:** 2021-07-02

**Authors:** Roberto Narcisi, Elena Jones

**Affiliations:** ^1^Department of Orthopaedics and Sports Medicine, Erasmus MC, University Medical Center, Rotterdam, Netherlands; ^2^Leeds Institute of Rheumatic and Musculoskeletal Medicine, The University of Leeds, Leeds, United Kingdom

**Keywords:** cartilage, bone, mesenchymal stem cells, osteoarthritis, musculoskeletal disorders

With increasing aging populations, musculoskeletal tissue disorders including osteoarthritis (OA) and fracture non-unions have become a significant healthcare challenge. The discovery of multipotential mesenchymal stem/stromal cells (commonly termed MSCs) in virtually all musculoskeletal tissues (da Silva Meirelles et al., 2006), combined with significant advances in industrial-scale cell manufacture (Bellani et al., 2020), have made musculoskeletal tissue regeneration a realistic opportunity. And yet, the regulatory approvals and clinical uptake of new therapies progress at a frustratingly slow pace. Apart from commercial considerations, a slow uptake of these therapies may be due to our limited understanding of the effects of mechanical, cellular, inflammatory and metabolic environments on MSCs, both before and after implantation. The aim of this Research Topic was to bring together recent research on cell-based bone and cartilage regeneration focussing on extrinsic mechanisms controlling cell fate.

The Research Topic is framed by two comprehensive review articles, with Goodman and Lin centered on bone healing and Greif et al. on the infrapatellar fad pad (IFP) as a source of MSCs and therapeutic target for osteoarthritis, a joint pathology influencing all joint tissues and often characterized by inflammation. Common for both reviews and the Research Topic in general is the focus on MSC potency enhancement, be it in elderly individuals or patients with co-morbidities. Goodman and Lin emphasize *ex vivo* cell pre-conditioning, hypoxia, genetic manipulations, and the use of biomaterials as potent modulators of cell function. Gene activated matrices combine the latter two factors and enable MSC migration and differentiation to occur in a tissue-relevant, three-dimensional manner. Greif et al. emphasizes the cross-talk between MSCs and M1 (pro-inflammatory) or M2 (anti-inflammatory) macrophages as an important determinant of the cellular and molecular milieu at the site of regeneration.

Oxygen levels are a known parameter influencing cartilage homeostasis and chondrocyte phenotype. In their study, Dennis et al. found that human chondrocytes self-assembled into cartilage sheets and cultured under physiologic conditions, were able to deposit an higher amount of extracellular matrix components, such as glycosaminoglycans, and become stiffer compared to cartilage sheets cultured in atmospheric oxygen levels. Along this line, in their review Lin et al. highlighted the importance of lysyl oxidase enzymes in regulating hypoxia-inducible factors in chondrocytes which, in turn, are key regulators of cartilage homeostasis. Lysyl oxidase enzymes can mediate cartilage integrity by regulating the expression of extracellular matrix-degrading enzymes matrix metalloproteinases and collagen cross-links, and have been recently associated to aging-associated cartilage degeneration. For this reason they are becoming very interesting potential targets for cartilage pathologies. Other extracellular matrix components of cartilage are Aggrecan and COMP. In their research, Caron et al., investigated the possibility of culturing progenitor periosteal cells under the influence of these matrix components aiming to sustain the chondrogenic differentiation process of those cells, which is often hampered by unwanted hypertrophic differentiation. What they found is indeed that those extracellular cartilage components have a potential favorable influence for the *in vivo* generation of cartilage for regenerative medicine applications since hypertrophy was significantly reduced when Aggrecan and COMP were added to the cell culture.

Bone regeneration with non-autologous MSCs is explored in Longoni et al., which presents a comprehensive *in vivo* investigation of segmental bone repair in a rat model. Using collagen-embedded, chondrogenically pre-differentiated xenogenic or allogenic MSCs, the authors demonstrate that bone bridging (*via* endochondral ossification) is “proportional to the degree of host-donor relatedness,” and that immune cell activation at both early and late stages of repair plays a major role in the healing outcome. These findings greatly improve our understanding of the immune cell mechanisms behind fracture non-union and fracture healing in general.

A controversial topic of platelet products (PPs), including platelet lysate (PL) for bone regeneration, is introduced in Goodman and Lin, where they call for a better standardization of PP/PL production and quality control. In this context, the study by Rikkers et al. presents an excellent example of good practice, where PL is thoroughly characterized for platelet purity as well as growth factor levels. The study describes the opposite effects of their PL formulation on chondrocyte expansion (enhancement) and re-differentiation (inhibition) thus emphasizing the complexity of chondrocyte responses to PL. Chondrogenesis enhancement for cartilage regeneration is also considered by Carroll et al., where the authors employ cell-seeded agarose hydrogels and a custom-designed oxygen sensor to compare the effects of different MSC sources (including IFP), cell density, and environmental oxygen tension on their chondrogenesis. The study opens up a possibility of sensor-monitored manufacturing of tissue engineered cartilage implants.

Sanjurjo-Rodriguez et al. compare MSCs derived from synovial fluid (SF) and subchondral bone (SB) of knee OA patients, and demonstrates a beneficial chondrogenesis-supporting gene expression in SF MSCs, particularly after joint distraction, a joint-preserving treatment for OA based on the correction of joint biomechanics. Together, the articles by Sanjurjo-Rodriguez et al. and Carroll et al. bring to the fore the effects of metabolic and biomechanical environments on MSC function.

Overall, in this Research Topic several important biological aspects regulating bone and cartilage phenotype at the cellular level have been discussed. Cell based-approaches for the treatment of musculoskeletal tissues are becoming more and more appealing for regenerative medicine and tissue engineering applications. A better understanding of the basic molecular processes governing cellular differentiation and commitment will allow us to explore new applications or further refine existing ones, in order to ameliorate the treatment of musculoskeletal disorders.

## Author Contributions

All authors listed have made a substantial, direct and intellectual contribution to the work, and approved it for publication.

## Conflict of Interest

The authors declare that the research was conducted in the absence of any commercial or financial relationships that could be construed as a potential conflict of interest.
